# Segmentation errors in macular ganglion cell analysis as determined by optical coherence tomography in eyes with macular pathology

**DOI:** 10.1186/s40942-017-0078-7

**Published:** 2017-07-17

**Authors:** Rayan A. Alshareef, Abhilash Goud, Mikel Mikhail, Hady Saheb, Hari Kumar Peguda, Sunila Dumpala, Shruthi Rapole, Jay Chhablani

**Affiliations:** 1 Department of Ophthalmology, McGill University, Montreal, QC Canada; 2 Department of Ophthalmology, King Abdulaziz University, Jeddah, Kingdom of Saudi Arabia; 3 Smt. Kanuri Santhamma Retina Vitreous Centre, L. V. Prasad Eye Institute, Kallam Anji Reddy Campus, Banjara Hills, Hyderabad, 500 034 India

**Keywords:** Artifacts, Errors, Ganglion cell algorithm, Ganglion cell inner plexiform layer, Spectral domain optical coherence tomography

## Abstract

**Background:**

To evaluate artifacts in macular ganglion cell inner plexiform layer (GCIPL) thickness measurement in eyes with retinal pathology using spectral-domain optical coherence tomography (SD OCT).

**Methods:**

Retrospective analysis of color-coded maps, infrared images and 128 horizontal B-scans (acquired in the macular ganglion cell inner plexiform layer scans), using the Cirrus HD-OCT (Carl Zeiss Meditec, Dublin, CA). The study population included 105 eyes with various macular conditions compared to 30 eyes of 30 age-matched healthy volunteers. The overall frequency of image artifacts and the relative frequency of artifacts were stratified by macular disease.

**Results:**

Scan errors and artifacts were found in 55.1% of the 13,440 B-scans in eyes with macular pathology and 26.8% of the 3840 scans in normal eyes. Segmentation errors were the most common scan error in both groups, with more common involvement of both segmentation borders in diseased eyes and anterior segmentation border in normal eyes.

**Conclusion:**

Segmentation errors and artifacts in SD OCT GCA are common in conditions involving the macula. These findings should be considered when assessing macular GCIPL thickness and careful assessment of scans is suggested.

## Background

Since the introduction of optical coherence tomography (OCT), OCT has become an integral part of the ophthalmological equipment, and in particular, for monitoring purposes. The selection of various imaging modes enables a broad variety of clinical applications. With the recent introduction of high-resolution spectral domain (SD)-OCT and the adaptation of this technique, visualization of distinct retinal layers has become feasible in vivo and has been shown to correlate well with histology [1–3]. This has numerous advantages; foremost, quantitative assessment of retinal layers over time facilitates longitudinal assessment of pathological processes and may become the standard for the assessment of effects of novel therapeutic substances. Several OCT scan modes are used to evaluate different aspects of the retinal pathology.

In recent years, the ganglion cell analysis (GCA) algorithm on Cirrus^®^ OCT (Carl Zeiss Meditec, Inc., Dublin, CA) has received particular attention and is gaining momentum as an important diagnostic tool in conditions involving the optic nerve and macula. The onboard Cirrus^®^ OCT GCA algorithm mode uses segmentation software for automated detection of the outer boundary of the retinal nerve fiber layer (RNFL) and the outer boundary of the inner plexiform layer (IPL) and provides measurements of ganglion cell inner plexiform layer (GCIPL) thickness, thereby allowing in vivo quantitative measurement of inner retinal layer thickness. It has emerged as a useful method for research and clinical practice, with applications spanning the evaluation of glaucomatous damage and progression, to indicating retinal neurodegeneration and being a biomarker for structural changes overtime [4–8]. Numerous reports have evaluated how retinal boundary artifacts can overestimate or underestimate macular thickness [2, 3, 9–18]. The occurrence of these artifacts may result in erroneous measurements of inner retinal structures. Recently we reported a 26.8% prevalence rate of artifacts in a normal population [19]. The prevalence, features and associated factors of artifacts involving the GCIPL in patients with macular involved retinal disease has not been previously evaluated, and no study has examined whether there is an alteration in the frequency of artifacts as the architecture of the retina changes in various macular diseases. It is thus beneficial to identify those scenarios in which artifacts and errors may occur, endangering accurate quantification and diagnosis in patients with conditions that impact the architecture of the macula.

The aim of this study was to assess the prevalence and magnitude of artifacts and errors in GCIPL segmentation in various pathologic entities of the macula and to compare those with scans obtained from a healthy population.

## Methods

Spectral-domain OCT (SD-OCT) data sets were gathered retrospectively from the patient cohort imaged with the GCA algorithm at L. V. Prasad Eye Institute, Hyderabad, India between January 2013 and July 2015. The local ethics committee approved the study and all patients gave informed consent prior to obtaining imaging. The macular scans were obtained from consecutive patients representing a spectrum of diseases involving the macula with no prior history of treatment. The study was conducted in accordance with the ethical standards stated in the 1964 Declaration of Helsinki.

One hundred and five eyes of 105 subjects with various macular conditions, including 15 eyes each presenting with diabetic macular edema (DME) secondary to type II diabetes, central serous chorioretinopathy (CSCR), idiopathic epiretinal membrane (ERM), dry age-related macular degeneration (AMD) and wet AMD, as well as an additional 30 eyes of retinitis pigmentosa (RP), were included. Inclusion criteria for this group were diseased eyes (as previously mentioned) with good quality scans (defined as a signal strength of more than 6), and a refractive error between −6.00 diopters (D) and +3.00 D spherical equivalent. The control group included 30 eyes of 30 healthy patients without any retinal or vitreoretinal interface abnormalities, with a good quality scan (signal strength of more than 6) and a refractive error between −6D and +3D [19]. One eye of each patient was included in the study. Subjects were excluded if they had coexisting ocular disease (e.g. uveitis, glaucoma or non glaucomatous optic neuropathy), a history of intraocular surgery, myopia >−6.00 D, hyperopia >+3.00 D or significant media opacities.

### OCT image acquisition and processing

Spectral domain OCT scans were obtained by using the Cirrus^®^ HD-OCT after pupillary dilation. The Macular Cube 512 × 128 scan protocol was used for all subjects. The protocol performs 128 B-scans and 512 A-scans per B-scan over 1024 samplings within a cube measuring 6 × 6 × 2 mm centered on the fovea. Images with signal strength <6 were considered of poor quality and discarded.

As described in our previous publications, the GCA algorithm was applied to the Macular Cube scans 9, 10 Briefly, the GCA algorithm identifies the outer boundary of the RNFL and the outer boundary of the IPL and provides measurements of GCIPL thickness. The average, minimum (lowest GCIPL thickness over a single meridian crossing the annulus), and sectoral (superotemporal, superior, superonasal, inferonasal, inferior, inferotemporal) GCIPL thicknesses were measured in an elliptical annulus around the fovea (dimensions: vertical inner and outer radius of 0.5 and 2.0 mm, horizontal inner and outer radius of 0.6 and 2.4 mm, respectively). The GCA algorithm measures the mean GCIPL thickness for each sector, compares them to the internal normative data- base of the device, and generates a thickness map, a deviation map, and a color-coded significance map. Measurements were displayed in green for normal range (P = 5%–95%), in yellow for borderline (1% < P < 5%), and in red for outside the normal range (P < 1%).

### Assessment of image artifacts

SD-OCT scans were obtained by using the Cirrus^®^ HD-OCT (Carl Zeiss Meditech, Dublin, CA) after pupillary dilation. The Macular Cube 512 × 128 scan protocol was used for all subjects. The protocol performs 512 horizontal B-scans comprising 200 A-scan per B-scan over 1024 samplings within a cube measuring 6 × 6 × 2 mm centered on the fovea. Images with signal strength <6 were considered of poor quality and discarded.

All 128 horizontal OCT B-scans acquired in the macular cube 512 × 128 protocol were examined by one evaluator (AG) with an intraclass correlation of 0.93 for the presence of artifacts.

Analysis of scans was performed as described in our previous publication [19]. Analysis of the images included infrared image, color-coded map and all individual 128 scans in an eye. Each B scan of the volume was reviewed to identify the potential cause of an artifact present in that B scan. Any form of segmentation error or artifacts was noted. Artifacts were identified and classified, and the number of B-scans containing artifacts was noted for each artifact type.

### Boundary line errors

#### Inner/outer segmentation line misidentification

The GCA algorithm software locates the outer boundary of the retinal nerve fiber layer (RNFL) presented as a solid purple line and the outer boundary of the inner plexiform layer (IPL) presented as a solid yellow line. Subsequently, all images acquired within each scan pattern (for example, 128 raster scans in Cirrus macular cube 512 × 128) were reviewed to check for segmentation breakdown in the inner and outer segmentation lines. The presence of a macular GCIPL segmentation error was defined as when these 2 lines were not located in the proper positions between the retinal layers in at least 1 cross- sectional image. If segmentation algorithm abnormality was noted in the outer boundary of the RNFL, it was recorded as inner segmentation line misidentification. If a segmentation abnormality was noted in the outer boundary of the inner plexiform layer (IPL), it was recorded as outer segmentation line misidentification. If a segmentation abnormality occurred in both lines in a single B scan, it was only recorded once as a segmentation error involving both borders of the GCIPL.

#### Missing segmentation line

Inner segmentation line absence was recorded in cases where a visible absence of the outer boundary of the RNFL line existed. In cases where an outer boundary of the inner plexiform layer (IPL) was missing, an outer segmentation line absence was recorded. Absence of both lines bordering the GCIPL was recorded as well.

#### Severity parameters related to boundary errors

To grade the severity of segmentation error, each scan was noted for segmentation deviation (inner or outer or both). Deviation of the segmentation line was classified into mild (<10 μm), moderate (10–50 μm) and severe (more than 50 μm). Each deviation, if present, was noted as upward or downward deviation.

Each error was further described according to its location on the scan. Each scan was divided into central 1000 μm, temporal 2500 μm and nasal 2500 μm. Segmentation error affecting the central part of the scan was specifically noted, as thickness measures from this subfield are commonly used to guide retreatment in patients with nAMD and DME. In addition, the central area of line scans may have a higher frequency of segmentation errors than more peripheral areas, as most patients might have fovea involving lesions with more disruption of the retinal ganglion cell RGC complex in this central zone.

Furthermore, the total scan area was divided in three zones: upper zone included 1st to 41st scan, central zone included 42nd to 84th scan and lower zone included 85th to 128th scan. Overall occurrence of the artifacts was described in these zones.

**Fig. 1:**
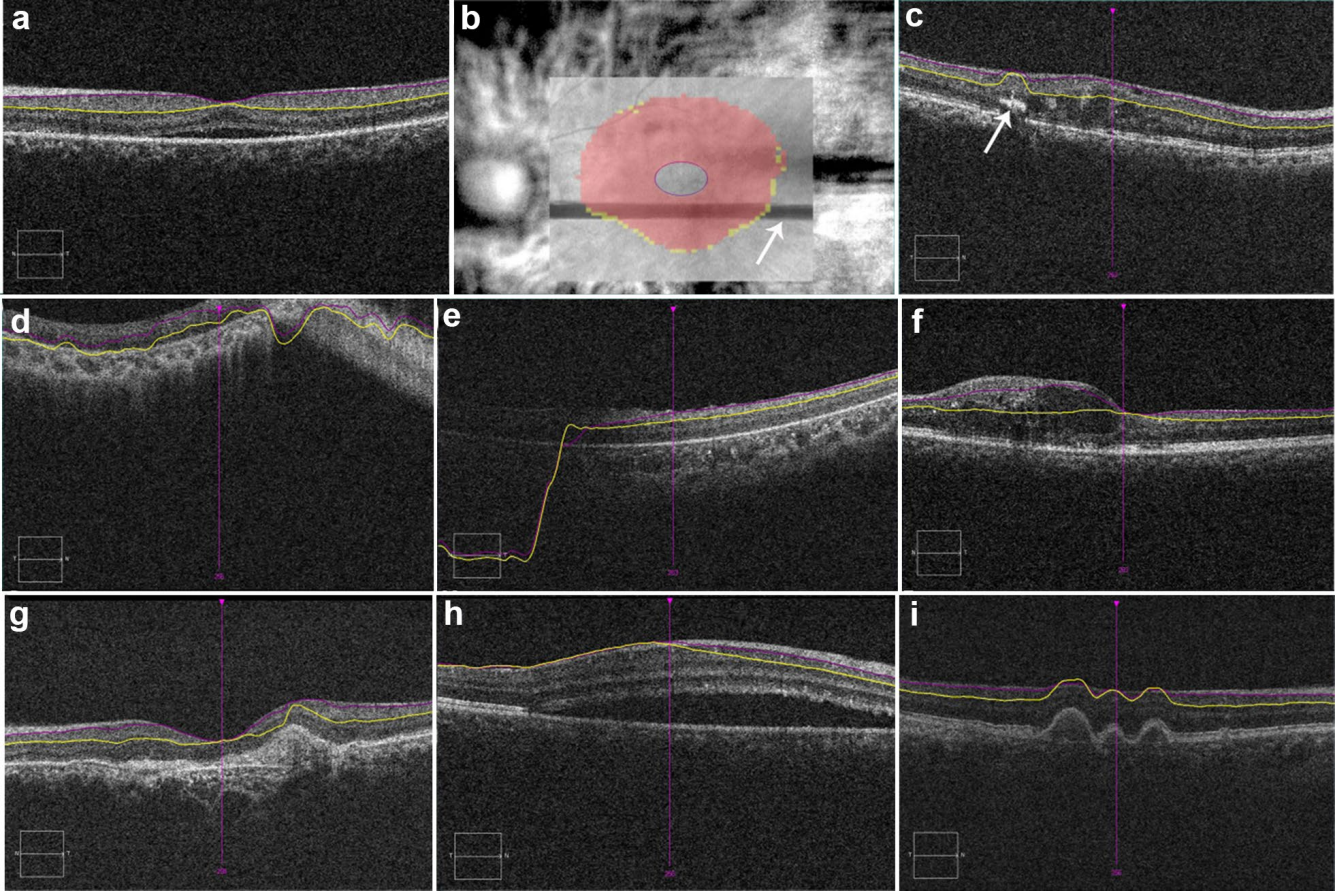
Composite figure shows various artifacts on ganglion cell analysis of Cirrus^®^ spectral domain optical coherence tomography. **a** Correct segmentation of ganglion cell inner plexiform layer in an eyes central serous chorioretinopathy (CSCR); **b** blink artifact on infrared image (*arrow* shows the missing information due to blink artifact); **c** outer segmentation artifact due to hard exudate (*arrow*) in diabetic macular edema; **d** misalignment of both segmentation lines along with mirror artifact in an eye with retinitis pigmentosa; **e** downward deviation of both segmentation lines; **f** outer segmentation artifact in an eye with diabetic macular edema; misalignment of both segmentation lines along with mirror artifact in an eye with scarred age-related macular degeneration (AMD) (**g**); CSCR (**h**); dry AMD (**i**)

Motion artifact was detected as misalignment of retinal vessels on rendered fundus infrared image. Figure 1 shows various artifacts in macular conditions. Other artifacts including defocus, out of register, shadowing, mirror artifact, and blink artifact were also evaluated.

### Statistical analysis

Descriptive statistics included mean and standard deviation. Statistical analyses were performed using commercial software (Stata data analysis and statistical software, version 12.1, StataCorp, College Station, TX). A P value of <0.05 was considered statistically significant.

## Results

### Eyes with macular related pathologies

The various retinal pathologies included were as follows: 30 eyes with RP, 15 eyes with wet AMD, 15 eyes with idiopathic ERM, 15 eyes with DME secondary to type II diabetes, 15 eyes with dry AMD, 15 eyes with CSCR. The mean age of the subjects was 49.54 ± 16.32 years (range 15–85). Gender distribution was 31 females and 74 males. Thirteen eyes were pseudophakic.

### Overall frequency of scan line artifacts

A total of 13,440 scans from 105 OCT macular cube scans of 105 eyes were reviewed. Artifacts were noted in 7410 scans of 13,440 scans, equivalent to a frequency of 55.1%. Artifacts were mostly observed in B scans of patients with RP 95.5% followed by neovascular AMD 64.47% and least in patients with ERM 19.7%.

### Frequency of artifacts by type of artifacts

For ganglion cell algorithm scans, misidentification of both lines was the most common segmentation line error (50%) observed. Segmentation line absence was the second most common segmentation line error (7.8%) observed, and it more likely involved the inner segmentation line (7.8%) Motion artifacts were the most common artifacts noted on IR images. Table 1 summarizes types of artifacts observed.

**Table 1:** Demographic variables and percentage and characteristics of segmentation errors identified using the ganglion cell algorithm analysis in macular diseases and healthy eyes

	Healthy eyes	RP	CSCR	Dry AMD	DME	Wet AMD	ERM
N (eyes)	30	30	15	15	15	15	15
Age (years)	56.3 ± 4.5	31.1 ± 11.81	37.6 ± 9.74	62.49 ± 11.49	68.0 ± 7.7	58.0 ± 14.2	53.6 ± 15.4
Mean signal strength	6.76 ± 0.81	8.36	8.17	6.60	6.80	6.46	6.86
Mean number of scans with error in one eye	35.84	122.9	52.6	20.5	61.5	75.8	25.26
Mean overall number of scans with errors (%)	26.8	95.5	41.0	20.5	48.0	64.47	19.7
Affected zone (%)							
Upper (1st–41st)	20	31.27	9.27	6.92	14.16	18.54	6.30
Central (42nd–84th)	30	31.32	16.04	9.01	18.33	25.98	5.20
Lower (85th–125th)	47	33.43	15.78	4.58	15.62	19.94	8.22
Affected scan area (%)							
Nasal 2500 μm	11.2	94.27	43.07	39.33	56.39	45.29	88.65
Central	16.8	86.06	79.48	46.15	77.42	85.02	88.15
Temporal 2500 μm	11.4	91.40	75.78	37.52	58.56	42.66	77.00
Layer affected (%)							
Inner	62	1.0	0.76	15.48	0	8.64	30.07
Outer	17.2	0.02	12.29	3.55	20.82	6.70	0
Both	20.79	99.59	86.94	80.96	78.95	84.65	69.92
Deviation (%)							
Upward	47.91	92.16	85.93	93.90	73.86	84.65	66.49
Downward	33.43	23.56	17.36	6.09	26.35	15.58	0
Both	18.65	2.52	0	0	0	0	33.50
Degree of deviation (%)							
Mild	38.6	77.81	88.97	94.41	98.59	94.91	96.04
Moderate	20.9	14.31	11.02	3.80	2.49	4.52	3.95
Severe	40.3	9.49	0	2.02	0	0.56	0
Type (%)							
Segmentation error	20.79	95.8	40.0	20.5	48.0	58.0	16
Segmentation loss	2.91	0.0.78	1.1	0.8	1	1.2	2
Defocus	6.70	4.4	0	0	0	0	0
Out-of-register	3.3	10.9	0	0	0	0	0
Shadowing	0	0.4	0	0	0	0	0
Mirror artifact	0	4.5	0	0	0	0	0
Blink artifact	0.09	0.4	0	0	0	0	0
Artifacts on IR image (%)							
Motion	53.3	33.33	6.6	20	26	23	6.6
Blink	0.09	6.66	0	0	0	0	0

*RP* retinitis pigmentosa, *CSCR* central serous chorioretinopathy, *AMD* age-related macular degeneration, *DME* diabetic macular edema, *ERM* epiretinal membrane

Percentage of B scans with inner and outer GCIPL misidentification was calculated for each of the disease states. Highest average percentage of an isolated outer GCIPL misidentification occurred in DME (20.8%), and CSCR (12.2%), whereas highest average percentage of an isolated inner retina misidentification occurred in wet AMD (30%), and dry AMD (15.4%). Furthermore, the highest average percentage of B scans with combined inner and outer GCIPL boundary misidentification occurred in RP (99.5%) and CSCR (86.9%).

When comparing the proportion of cross-sectional retina scans with errors, nearly all artifact types were more common in the center 1000 micron area of the scan in all disease categories but RP and ERM, where most of the errors were located in the nasal 2500 μm area of the scan. When reviewing cross-sectional scans with errors, location of errors was not correlated with location of pathology. Upward Deviation of the segmentation line occurred more commonly then downward segmentation line deviation. Upward deviation occurred more in RP (92.1%) and dry AMD 93.9%. Downward deviation occurred more frequently in DME 26.35% and RP (23.5%). Among all disease categories, ERM had the least amount of deviations. When comparing the degree of segmentation line deviation, mild deviation was more often noted in all disease groups.

### Causes of artifacts in disease categories (Table 2)

#### Retinitis pigmentosa

The majority of errors observed was misidentification of layers secondary to hyper-reflectivity of the RNFL 92.36%, where the algorithm misinterpreted the highly reflective RNFL as the inner border of the GCIPL, other causes included defocus errors 6.47%, floater 0.46% and out of register scans 5.10%.

**Table 2:** Various identifiable causes of segmentation errors in various macular conditions

	RNFL hyper-reflectivity	SRF	PED	Floater	Degraded image	Inner retinal thickening	Hard exudates
CSCR	20%	12%	5.7%	0	0	0	0
RP	92.36%	0	0	0.46%	0	0	0
ERM	15.10%	0	0	0	0	0	0
Dry AMD	3.95%	0	14.0%	0	3.48%	0	0
DME	10.67%	0	0	0	0	27.86%	4.42%
Wet AMD	12.5%	11.45%	28.69%	0	0	6.14%	0

*RP* retinitis pigmentosa, *CSCR* central serous chorioretinopathy, *AMD* age-related macular degeneration, *DME* diabetic macular edema, *ERM* epiretinal membrane, *PED* pigment epithelial detachment, *SRF* subretinal fluid

#### CSCR

Hyper-reflectivity of the RNFL was also the most common identifiable element causing artifacts in 20% of scans. Furthermore, 12% were caused by subretinal fluid, 12% as a result of pigment epithelial detachments (PED) while 5.7% were other morphological elements.

#### ERM

Again, high reflectivity of the RNFL was identified as the cause of artifacts in 15.1% scans. The epiretinal membrane itself was the cause for artifacts in 3.6% scans.

#### Dry AMD

Pigment epithelial detachment was a cause in 14% of scans. Degraded image was also observed in 3.48% scans.

#### DME

Among DME eyes, inner retinal thickening was the cause in 27.86% scans, whereas hyper-reflectivity of the RNFL and hard exudates were morphological elements that resulted in artifacts in 10.67 and 4.42% scans respectively.

#### Neovascular AMD

Identifiable causes included RNFL hyper reflectivity in 12.5% scans, subretinal fluid in 11.45% scans, PED in 28.69% scans and inner retinal thickening in 6.14% scans.

### Normal healthy eyes

We reported these results in our recent publication [19]. Briefly, A total of 1029 (26.8%) out of total 3840 scans had scan errors. Misidentification of the inner GCIPL boundary was most frequent (62%). Upward Deviation of the segmentation line (47.91%) and severe deviation (40.3%) were more often noted. Artifacts were mostly located in the central scan area (16.8%). The average number of scans with errors per eye was 34.3% and was not related to signal strength on Spearman correlation (P = 0.36) (Table 1).

## Discussion

In ophthalmic imaging, the results of image segmentation can assist with locating layer pathologies, measuring tissue and layer volumes, studying anatomical structure and diagnosing several disorders. For example, retinal tissue segmentation for delineation of anatomical structures from OCT images plays an important role in several scenarios, this is particularly relevant in the evaluation of neurodegenerative disorders [20], where one can characterize morphological differences between subjects based on volumetric analysis of GCIPL, RNFL and outer retinal layers [3, 9, 11]. However, these are valid only if the results of image segmentation are correct. Image artifacts such as the presence of motion, retinal layer misidentification, can cause classification errors in the results of image segmentation. As a result, image segmentation is still a challenging task in ophthalmic image processing.

When compared to normal eyes [19], our results indicate that the accuracy of macular GCIPL thickness measurements may be largely influenced by the presence and severity of macular disorders. In contrast to OCT technology development which has been a field of active research since 1991, OCT image segmentation has only been more fully explored during the last decade. Segmentation, however, remains one of the most difficult and at the same time most commonly required steps in OCT image analysis. No typical segmentation method exists that can be expected to work equally well for all tasks [2]. In this paper, we tried to cite most related works from 1997 to 2012, however, this is in no way complete. It should also be noticed that the number as reported in tables cannot be used for direct comparison of the relative performances, since different settings are utilized in each method.

With the Zeiss Cirrus^®^, the measurement of inner retinal thickness is determined by inbuilt protocols of measurements. Although different machines may have a different number of artifacts, it appears that SD-OCT has reduced errors in automated retinal delineation. It has been reported that a low frequency of errors on the Cirrus^®^ OCT compared to the frequency of errors of other OCT instruments when evaluating patients with retinal disease [2, 16]. Errors linked to post-image processing and OCT instrument software frequently were observed and most commonly involved misidentification of the retina layers. More convincingly, Hwang et al. evaluated GCA related artifacts in glaucomatous and normal eyes without macular abnormalities and identified segmentation errors in 9.7% of eyes. In their study, the most prominent features of segmentation errors were: (1) they affected both the inner and outer segmentation lines (2) they were located at the nasal quadrant (centered to the fovea), (3) they were diffuse. Furthermore, the presence of a segmentation error was associated with a higher degree of myopia. In contrast to their study, and to shed some light on the impact of macular diseases on segmentation errors we strictly included eyes with macular disorders. While evaluating the RNFL and GCIPL of several diseased eyes, we noted misidentification artifacts involving the inner and outer GCIPL segmentation line. Overall, misidentification of both boundary lines of the GCIPL were most common (50%), followed by outer boundary misidentification, which occured in 2.9% of eyes.

Recent studies have investigated the macular alterations associated with both dry and wet AMD at the level of the inner retinal layers using OCT devices [21–23]. Zucchiatti et al. [22] reported that macular GCIPL involvement displayed by thinning is present in both types of AMD using Cirrus^®^ OCT. They also theorized that inner retinal morphology alteration might be helpful when considering treatment options. For this reason, artifacts and errors might be of additional value to inner retinal topographic evaluation and decision-making strategies. Ho et al. compared various OCT devices in patients with various retinal conditions and showed that Cirrus^®^ HD-OCT has the lowest percentages of any type of artifacts for patients with wet AMD [2]. In our study, more scan artifacts were observed in eyes with wet AMD compared to dry AMD, 64.4 and 20.5%, respectively.

Using the Cirrus^®^ OCT, Ho et al. [2] examined 15 patients with ERM and reported improper central thickness measurements (10%) secondary to artifacts. Using the GCA algorithm in 15 eyes with idiopathic ERM in our study, errors occurred in 19.7%. Additionally, in their study, eight patients had DME and diabetic retinopathy of which 25% of B scans were with outer retina misidentification and 23% of B scans with inner retina misidentification [2]. This is higher than what we observed in our study where none of the B scans had isolated inner GCIPL segmentation line misidentification (boundary line artifact), and 20.8% of B scans showed isolated outer GCIPL misidentification. However, B scans with inner and outer GCIPL boundary misidentification were seen in 78.9% of B scans. The higher number of patients in our study could explain this. The frequency of GCIPL boundary artifacts in eyes with DME was 48% in our study. Furthermore, the segmentation algorithm misinterpreted the hyper-reflective intraretinal areas representing hard exudates to correspond to the outer IPL boundary, missing correct alignment of the outer IPL border. This led to the wrong estimation of the GCIPL thickness, as calculated by the software.

Our results show that RP patients exhibited the greatest mean number of errors across all types of pathology, despite a better mean signal strength (8.36) when compared to other conditions. This could be related to fixation difficulties and pan-layer architecture changes in the retina. Ho et al. used the Cirrus^®^ Cube 512 × 128 and reported that Stargardt disease caused the greatest percentage of significant OCT examination error. Although the pathophysiology of RP is different from Stargardt disease, the progressive loss of vision in both diseases may be related to the macular involvement, which may cause a higher frequency of artifacts.

The vast majority of patients exhibited inner segmentation error, and this was common across all pathologies. Similarly, deviation was mostly in the upward direction rather than in the downward direction. Outer border misidentification is likely to be higher in eyes presenting in pathology in the outer retinal layer, such as subretinal fluid in CSCR or wet AMD, as well as drusen in dry AMD. These can have significant clinical implications, particularly in the follow-up of those patients. The current GCA algorithm does now allow manual correction of inner and outer border misidentifications.

The presence of morphologic features, such as fluid between the retina and the RPE or fluid within the retina, may confound the segmentation algorithms that attempt to identify the inner retinal boundaries. In contrast, eyes with a diagnosis of dry AMD with no morphological elements were less likely to have errors in retinal segmentation. This observation increases confidence in the use of quantitative OCT data in studies of dry AMD, but raises concerns regarding its use in the examination of neovascular AMD, DME and RP. Furthermore, the impact of these conditions on GCIPL measurement should be considered when interpreting the GCA in glaucoma patients with macular co-morbidities.

Recently published OCT segmentation reports focus on improving precision and accuracy, reducing the required processing time and increasing the understanding of the various errors associated with these segmentation algorithms [13, 21, 24–26]. Current imaging modalities are moving research in the direction of layer and volume segmentation, as well as 3-D rendering and visualization. It is therefore important to develop robust methods that are capable of dealing with pathologic cases in OCT imaging.

The limitations of this study include a small sample size in each group. The inaccuracy of the macular GCIPL thickness could not be assessed because the software does not permit manual correction when an artifact is detected. We did not longitudinally assess if the frequency of these artifacts change with time or in response to treatment. Although this study examined eyes across a broad spectrum of macular disorders, the results obtained from this study can only be generalized to this subset of patients undergoing this specific imaging protocol. We did not evaluate effect of vision, fixation, or the grade of cataract on artifacts. Because of the small sample size in each disease, valuable correlation between retinal thickness, signal strength and GCIPL thickness could not be studied. We did not evaluate the effect of repeat scans on artifacts. Nevertheless, to the best of our knowledge, this is the only study reporting the frequency, type and distribution of artifacts, as well as the morphological elements that may lead to artifacts using the GCA algorithm. Finally, we have not evaluated the effect of repeat scans on artifacts.

In conclusion, the GCA algorithm provides a significant advance in our ability to image and assess the inner retina. Nevertheless, there are multiple errors associated with the segmentation and identification of retinal layers. In addition, multiple artifacts can be noted both in pathological and normal eyes. Clinicians must pay attention to these errors and factor them into their decision making process.

## Abbreviations

GCA: ganglion cell analysis
GCIPL: ganglion cell inner plexiform layer
RP: retinitis pigmentosa
CSCR: central serous chorioretinopathy
AMD: age-related macular degeneration
DME: diabetic macular edema
ERM: epiretinal membrane
